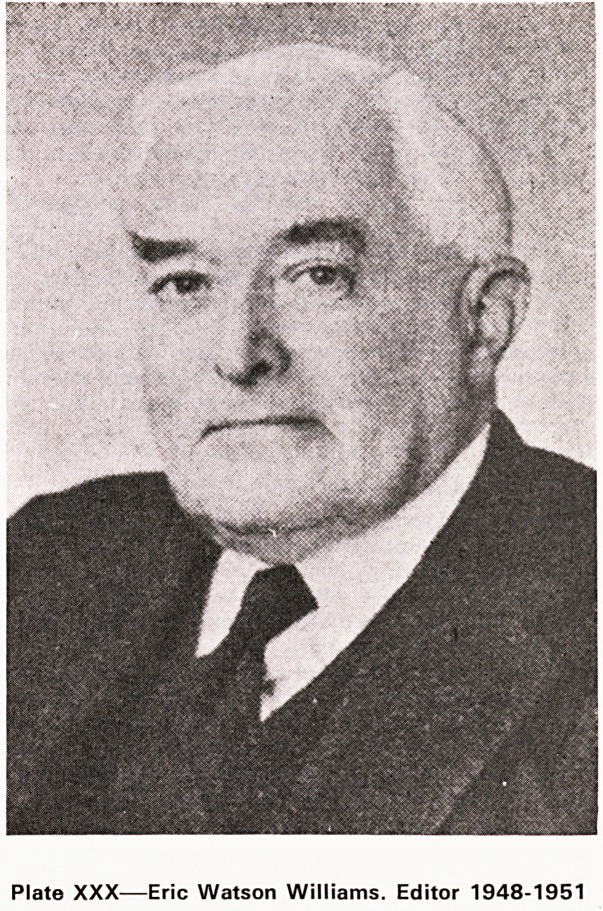# Medical Journalism in the West of England 1883-1970
*The Long Fox Memorial Lecture, delivered in the University of Bristol on 19th November 1970.


**Published:** 1974-07

**Authors:** N. J. Brown

**Affiliations:** Consultant Pathologist, Southmead Hospital, Bristol


### Medical Journalism in the West of England 1883-1970*

By
**N. J. Brown, M.B., F.R.C.P., F.R.C.Path.**
Consultant Pathologist, Southmead Hospital, Bristol

I would first like to thank the University for the honour they have done me by asking me to deliver this Long Fox Memorial Lecture and to say that it is with a great sense of unworthiness that I attempt the task. Since this annual lecture was instituted in 1904 a great many distinguished men have spoken with eloquence on subjects of which they were masters. I would like to suggest that tonight the distinction lies less in the speaker than in the subject, for in this city we have a unique medical treasure—a local medical journal with an unbroken record of publication since the year 1883. I refer, of course, to the Bristol Medico-Chirurgical Journal which is believed to be the oldest provincial medical journal with a continuous record of publication, in this country today. It has been my privilege to have been associated with this journal in one capacity and another for the past fifteen years and now, as only its ninth editor in nearly 90 years, I would like tonight to tell you something of its history, of the people who have been concerned with its production, of those who have written in it and what they have had to say; of how it forms a fascinating record of medical thought, progress (and politics), in Bristol and the West Country over the years. I would like, as it were, to be merely its mouthpiece and to let the Journal speak for itself and give its own unique record of medical history in this part of the world over the past 87 years.

It is customary at the outset of this lecture to pay some tribute to the memory of Dr. Edward Long Fox but, with your permission, on this occasion I would like to postpone this to a later part of the talk, the reason being that there is such a close association between Dr. Fox and his family and the Journal that I shall not be able to speak of one without the other and it may be that his life and character will come through to you more clearly in the passing references than it might do in the traditional, sometimes rather perfunctory, phrases which a lecturer often feels obliged to utter before getting down to his real subject.

Although my main theme will be the story of the Bristol Medico-Chirurgical Journal, I shall have a few words to say about other medical journals past and present which have appeared from time to time in the West of England.

**Bristol in 1883**

The Bristol Medico-Chirurgical Journal first appeared in 1883. Let us look at the state of medical affairs in Bristol at that time. The Infirmary (founded in 1737) had been Royal for 33 years and consisted of what we now call the 'old building' only. The General Hospital (founded in 1831) was nearly a hundred years younger and the Children's Hospital (founded in 1886) was new. There was also the Eye Hospital which had been opened in 1810.

There was, of course, nothing remotely resembling the National Health Service as we know it today. Those patients who could, paid; those who could not depended on charity, receiving their treatment not only from the hospitals but also from such institutions as the Clifton Dispensary or the Eye Dispensary in Orchard Street. The physicians and surgeons gave their services to the hospitals free, making a living entirely from their paying patients. The physicians used many drugs but few of them were very effective. Antisepsis had been in general use in surgery for only about fifteen years. The most commonly used anaesthetic was chloroform, although nitrous oxide and ether were also available. New bacteria were busily being discovered by Pasteur and others. Robert Koch had demonstrated the tubercle bacillus only the previous year and it was to be another twenty years before the spirochaete of syphilis came to light. X-rays were not yet discovered and many diseases known today were still undescribed. The medical profession was held in high esteem; many of its members were individualists and men of strong character. Nursing was becoming established as a profession but women doctors were a rare novelty.

The Bristol Medical School was fifty years old. It was affiliated to Bristol University College which had been founded in 1876. The medical students studied anatomy and physiology at University College and then pursued their clinical studies at either the Royal Infirmary or the General Hospital, which were virtually separate teaching establishments. Except that University College became the University of Bristol in 1909, the same system persisted up to the time when I myself became a student here in the 1930s.

* *The Long Fox Memorial Lecture, delivered in the University of Bristol on 19th November 1970.*

**Origins of the Journal**

Against this background the Bristol Medico-Chirurgical Society had been founded in 1874. In 1878 the Society produced its first volume of Transactions, a record of five years work. A second volume was planned for 1882 but there was said to be not enough material. Instead it was proposed to the Society's committee by L. M. Griffiths, seconded by Greig Smith that a journal should be started but the committee turned the idea down. A month later a General Meeting of the Society reversed the committee's decision and the Journal was born. Greig Smith who had previously helped to edit some Infirmary reports in 1879 was appointed the first Editor. As I have already indicated, he was the first of the nine editors the Journal has so far had and I propose to take each editor in turn, surveying the contents of the Journal during each of the nine 'reigns'. Nine sections may seem a lot but may perhaps be more acceptable than a consideration of each of the 85 volumes!

**James Greig Smith (Editor 1883-1891)**

James Greig Smith (Plate XIII) was a Scot who had graduated in Arts and Medicine at Aberdeen. He came to the Royal Infirmary as Assistant House Surgeon and eventually became a Surgeon there. He was a distinguished surgeon, a rapid diagnostician and a skilful and careful operator. He talked to himself incessantly while operating but did not like anyone else in the theatre to talk much. He published a book on abdominal surgery which ran to five editions. A strong character, scrupulously exact in his own work and impatient of lower standards in others, he became lecturer and then Professor of Surgery in Bristol University College. He was a clever artist and used this talent in recording pathological conditions as well as drawing caricatures of his friends. He was 29 and had been qualified for only seven years when he became editor; he died of lobar pneumonia when only 43 years of age. An operating theatre at the Infirmary was named in his memory.

Plate XIII—James Greig Smith. Editor 1883-1891

JULY, 1883.

*Vol. I.*] [*No.* 1.

THE

BRISTOL

MEDICO-CHIRURGICAL

JOURNAL

A Journal of the Medical Sciences for the West of England and South Wales

PUBLISHED UNDER THE AUSPICES OF

*THE BRISTOL MEDICO-CHIRURGICAL SOCIETY*

EDITED BY

J. GREIG SMITH

M.A., F.R.S.E.

"Scire est nescire, nisi id me Scire alius sciret."

BRISTOL: J. W. ARROWSMITH

LONDON: J. & A. CHURCHILL, 11 NEW BURLINGTON STREET

*Price Two Shillings and Sixpence.*

Plate XIV—Cover of Vol. I, No. 1

He arranged for a circular to be sent to every practitioner in the West of England and South Wales soliciting support for the new Journal and so in July 1883 there appeared Volume I No. 1. Let us dwell on this first number a little.

### The first number

The cover (Plate XIV) carries a sub-title 'A Journal of the Medical Sciences for the West of England and South Wales' and bears a latin epigram *Scire est nescire, nisi id me Scire alius sciret.*

This quotation, said to be from Lucilius (Smith 1900) became the Journal's motto and appeared at the front of each issue up to 1952. My classicist friends tell me that literally it means 'To know is not to know unless someone else knows that I know' or, more freely translated, 'Knowledge is ignorance unless that knowledge is made known to others'. The first number contained no less than 142 pages of text and its price was two shillings and sixpence.

On opening this copy the advertisements are immediately fascinating: the first is for a firm of Bristol opticians still in existence today, the next for a firm of 'Tailors and Habit Makers' in Castle Street proclaiming

> 'Every first order is drafted by a foreman cutter of recent London experience. . . . The best proof of the care taken is the fact that in the past season no ordered garment has been rejected for misfit . . . they earnestly invite application for patterns and prices—or a trial order—for comparison; no fair comparison, however, can be made on cheap trousers only, which it is a common trade practice to mark for lure.'

We are immediately in a different world. There follows an advertisement for the Medical School (Plate XV), listing 21 lecturers; as there are now 445 teachers in the Faculty of Medicine, perhaps the students today are expected to know twenty times as much! In the advertisement for the Royal Infirmary which follows we notice Edward Long Fox among the Consulting Physicians and of particular interest to me is the statement that 'Senior Students, appointed quarterly, perform all the post mortem examinations'. After similar notices of the clinical experience available at the General Hospital, The Eye Hospital and the Eye Dispensary there are set out the officers of the Bristol Medico-Chirurgical Society and a list of subscribers in which it is interesting that only 14 of some 300 were in fact from South Wales.

University College, Bristol.

### MEDICAL SCHOOL.

The **WINTER SESSION** will commence on **MONDAY, OCTOBER 1st.**

### Lecturers :

*Medicine*—W. H. SPENCER, M.A., M.D. Cantab., and E. MARKHAM SKERRITT, M.D. Lond., B.A., M.R.C.P.

*Surgery*—N. C. DOBSON, F.R.C.S.

*Anatomy*—F. R. CROSS, M.B. Lond., F.R.C.S.

*Practical Anatomy*—Demonstrator, W. H. HARSANT, F.R.C.S.

*Physiology*—R. SHINGLETON SMITH, M.D. Lond., B.Sc., M.R.C.P.

*Chemistry*—T. COOMBER, F.C.S.

*Hygiene*—D. DAVIES, M.R.C.S.

*Midwifery and Diseases of Women*—J. G. SWAYNE, M.D. Lond., and A. E. AUST LAWRENCE, M.D.

*Medical Jurisprudence*—R. EAGER, M.D. Lond., and A. J. HARRISON, M.B. Lond.

*Pathology and Morbid Anatomy*—W. H. SPENCER, M.A., M.D. Cantab., and E. MARKHAM SKERRITT, M.D. Lond., B.A., M.R.C.P.

*Operative Surgery*—W. P. KEALL, M.R.C.S.

*Practical Surgery*—A. W. PRICHARD, M.R.C.S.

*Materia Medica and Therapeutics*—J. E. SHAW, M.B. Ed.

*Practical Physiology and Histology*—G. F. ATCHLEY, M.B. Lond.

*Demonstrator of Physiology*—G. MUNRO SMITH, M.R.C.S., L.R.C.P.

*Botany*—A. LEIPNER.

*Practical Chemistry*—T. COOMBER, F.C.S.

*Comparative Anatomy*—Prof. W. J. SOLLAS, M.A. Cantab.

*Medical Tutor*—J. F. EVANS, M.B., C.M. Ed.

Composition Fee for Lectures, 60 guineas.

HOSPITAL PRACTICE may be attended either at the Bristol Royal Infirmary or at the Bristol General Hospital.

FEES (including Clinical Lectures)—Infirmary: Perpetual Medical and Surgical Practice, 20 guineas each, or in one payment, 35 guineas. Hospital: Perpetual Medical and Surgical Practice, £20 each.

SCHOLARSHIPS AND PRIZES.—Numerous valuable Scholarships and Prizes are offered by the Medical School, and by the Infirmary and the Hospital.

*For Prospectuses and particulars apply to*

E. MARKHAM SKERRITT, M.D., *Hon. Sec.*

Medical School, University College, Tyndall's Park, Bristol.

**Plate XV—From Vol. 1 (1883)**

The first two papers are concerned with the contagiousness of phthisis, Shingleton Smith (Royal Infirmary) saying that the disease is contagious and Markham Skerritt (General Hospital) arguing that the case was not proven. It may seem strange to us that there could be any doubt in the matter but at that time although Koch had recently discovered the tubercle bacillus and William Budd nearly twenty years earlier had declared the disease to be infectious, not everyone accepted that the bacillus was the cause. As Skerritt says 'this organism may be associated with tubercular lesions without necessarily causing them—just as smoke is associated with fire and is therefore of value in its diagnosis, though it does not cause it'. As a confirmed General Hospital man myself I am sorry that Skerritt backed the wrong horse but he was a big enough man to imply later that he was wrong (Skerritt 1892).

Next comes an article by Munro Smith on 'The Cardiograph in Medicine'. This at first seems surprising until we realise that it is not the *electro*cardiograph that he refers to but what we would probably call a sphygmograph. A number of case reports follow describing congenital syphilis, lupus vulgaris and a number of surgical conditions and then two cases of spinal cord compression due to sarcomatous growths by Edward Long Fox whom we commemorate tonight, illustrated by a beautiful drawing (Plate XVI) which is the Journal's first illustration. A few pages further on there are several drawings by the Editor, Greig Smith, himself.

Towards the end of this first number we come to Notes on Books—anonymous reviews, scathing and ruthless—for example:

> 'It is a large and pretentious volume . . . but the type is remarkably large, and the amount of matter in each page is consequently not great. Dr. —— distinctly states that his book is especially intended

Sarcomatous Growth compressing the Spinal Cord.

**Plate XVI—The Journal's first illustration (1883)**

for his medical brethren. Were it not for that statement we should have been disposed to think it was principally directed to catch the popular ear. . . . We fear that Dr. ——'s work will not add much to our knowledge or conduce greatly to his own reputation.'

Would that people wrote reviews like that nowadays! This is followed by extracts from contemporary books and journals including an account of an unusual operation—removal of the gall bladder—and a description of an ingenious device for trapping tapeworms. More advertisements complete the number—Life Insurance, Mineral Waters, and a wine merchant offering sherry at 16-18 shillings per *dozen.*

### The 1880s

We must move on to Volume II (1884). This contains two articles by Edward Long Fox. In discussing the nature and treatment of Chorea many of his observations would still be valid today but it is interesting that he regards two examples of the congenital form as having been caused by the mother being frightened, in one case by a snake, during pregnancy. The other paper is an account of Spontaneous Cure of Spina bifida followed by Hydrocephalus. In this case too the mother had been frightened by a 'furious fowl'. The article is illustrated by the first photograph to appear in the Journal (Plate XVII). Photogravure at that time was in its infancy and the illustration consists of an ordinary photographic print pasted on to the page.

**Plate XVII—The Journal's first photograph (1884)**

Volume III (1885) is absolutely fascinating. There are two more papers by Edward Long Fox, one on 'Enlargement of the Spleen' and the other on 'Mangana Water'. A Mr. Keevill of Clifton had devised an effervescing mixture based on analysis of the famous mineral water of Cransac in the South of France. Having described the preparation, Fox goes on 'It would not be becoming in me to say anything that might seem to be a testimonial of an article of commerce but I venture to ask my professional brethren to give some trial to this convenient therapeutical agent, especially in chronic congestion of the liver, and I think they will agree with me in finding it useful.' As we shall see, some of his professional brethren were less inhibited. In the same volume we find an article on 'Explosive Drugs'—an account of injuries caused by the explosion of compounds ordered in physicians' prescriptions—and a paper advocating cremation. The latter, by J. G. Davey of Clifton, takes a somewhat bizarre turn. After giving an account of trance-like states that might be mistaken for death, the author points out the terrible danger of being buried alive which to him seems a disaster to be avoided at all costs. Almost unbelievably the argument unfolds until the triumphant conclusion is reached that 'with cremation no such catastrophe could ever occur'!

Then there is this little gem:—

'Case of Attempted Suicide by Precipitation from Clifton Suspension Bridge'
By J. Fenton Evans

—a report by the House surgeon at the B.R.I. of a girl aged 22 who was the only person so far to have jumped from the bridge and survived. He thought this was probably due to 'the nature of her clothes which are said to have been inflated from below and thus delayed the descent'. The Suspension Bridge, built in 1864, was then only 21 years old.

Passing on through the 1880s, advertisements for the Children's Hospital begin to appear and descriptions of local Health Resorts were published. Of Clifton it was said 'There is plenty of ozone in the atmosphere except when the wind blows from over Bristol, and if people do not live very fast, they are not in such a hurry to die as in some more bracing places'. It was only equalled by Budleigh Salterton for asthmatics and one wealthy patient who had been nearly everywhere declared that 'The north side of the Old Mall at Cilfton was the safest place in Europe', and of Weston-super-Mare 'The sea recedes a considerable way in Weston Bay, leaving exposed to the sun and the sea-breezes a large tract of mud . . . this mud is one of Weston's most important features; for the Atlantic bree_es blowing across it catch up a large amount of ozone and iodine, and then discharge their load into the lungs of visitors and residents alike, making the weak strong and the strong feel as giants.'

In 1891 Greig Smith retired from the post of Editor and became President of the Medico-Chirurgical Society, giving his presidential address on 'Modern Medical Journalism'. He deplored a lot of articles then being written, for reasons which would still hold good today. He made a plea for a national journal, heavily subsidised, printing only papers of the highest standing, and free from all considerations of making money.

Our own Journal meanwhile in its short life had made a great deal of money, so much that there were suggestions that it should be given to charity. On the proposal of Greig Smith however it was decided to start a library fund and so there came into being the Society's library which was eventually handed over to the University and became the nucleus of the fine University Medical Library which we enjoy today.

**Lemuel Matthews Griffiths**

Although he never became Editor, special mention must be made of L. M. Griffiths (Plate XVIII) whose services to the Journal were second to none. He had

**Plate XVIII—L. M. Griffiths. Assistant Editor 1883-1900**

been Assistant Editor from the start and continued so until 1900. A Bristolian, trained at Bristol Medical School he became a general practitioner in Hotwells. He was a born librarian and a great lover of books, every room in his house, including the bathroom, being full of them. One room was devoted entirely to Shakespeare on whom he was an authority. He collected and indexed cuttings and set out to collect a portrait of each character in the Dictionary of National Biography, his collection being eventually bequeathed to the National Portrait Gallery. A

**Plate XIX—From Vol. V (1887)**

**Plate XX—From Vol. XIII (1895)**

staunch Churchman he did much to entertain and educate the poor people of St. Jude's. He died in 1924 aged 79.

From 1886 onwards the Journal featured 'Scraps—picked up by the Assistant Editor'. These were humorous stories and amusing and entertaining extracts from wide sources; some were from French journals—in French—and there were occasional Latin verses. This is supposed to be a serious lecture but one cannot resist some examples of medical humour of the period.

*Another Hermaphrodite*

Among the replies to an advertisement of a musical committee for 'a candidate as organist, music-teacher, etc.' was the following one. 'Gentlemen—I noticed your advertisement for an organist and music teacher, either lady or gentleman. Having been both for several years, I offer you my services.'

*Cooling Medicine*

From diary of a young lady crossing the ocean: 'At 8 o'clock in the evening took a pill. At six in the morning passed an iceberg.'

*Poor Babies*

Advert. for new feeding bottle: 'When the baby has done drinking, it must be unscrewed and laid in a cool place, say under a tap.'
In similar vein—'if the baby does not thrive on fresh milk it should be boiled.'

*Too knowing*

Pompous physician (to patient's wife): 'Why did you delay sending for me until he was out of his mind?' Wife: 'Oh, doctor, while he was in his right mind he wouldn't let me send for you.'

### NESTLE'S FOOD

**AN ENTIRE DIET FOR**

### Infants, Children, and Invalids.

"Renders valuable assistance in Wasting Fever."
"Beneficial as a diet in severe cases of Typhoid."
"Invaluable in Cholera Infantum."

*Pamphlet containing Extracts from Standard Medical Works in which the above Testimony is given sent free, with Sample Tin, on application to*

H. NESTLÉ, 48 Cannon Street, London, E.C.

**The attention of the Medical Profession is also drawn to**

**NESTLÉ'S**

**SWISS**

(CONDENSED)

**MILK**

**Which, through its RICHNESS in CREAM and UNIFORMITY in QUALITY, has obtained the Largest Sale in Great Britain. It can be used for all purposes of FRESH MILK.**

*Samples to Members of the Medical Profession only sent free on application to*

**H. NESTLÉ,**
**48 Cannon St., London, E.C.**

**Plate XXI—From Vol. XIV (1896)**

**Plate XXII—From Vol. XV (1897)**

Other features at this time were 'Preparations for the Sick' which continued until 1921, 'Periscope of Medical and Surgical progress'—review articles on selected subjects which commenced in 1886—and Obituary Notices. The first of these appeared in 1891 for Frederick Brittan, the first president of the Medico-Chirurgical Society. And always there were the advertisements which brought in the money (see Plates XIX-XXII).

**Robert Shingleton Smith** (Editor 1892-1912)

The Editor for the next twenty years was to be R. Shingleton Smith (Plate XXIII), a graduate and gold medallist of London University from King's College who was to become one of the best known consulting physicians in the provinces. He became lecturer and then Professor of Medicine in Bristol. He was one of the founders of the Medico-Chirurgical Society, its first secretary, and its president in 1888-9 and the author of the first paper in the first number of the Journal. An excellent teacher he was also a keen antiquary, gardener and astronomer.

**Plate XXIII—R. Shingleton Smith. Editor 1892-1912**

During the 1890s the Journal continued to catalogue and record clinical cases and pathological findings and articles on a wide variety of subjects appeared. Comparative pathology, based on animals at the Clifton Zoo, crept in; there were intriguing accounts of 'Unusual operations on Lunatics', 'The Use of

**Plate XXIV—Device for quantitative estimation of knee jerk. Vol. X (1892)**

Alcohol in Medicine' and 'Diseases of Sewer Workers' with special reference to Clifton. It is also clear that Bristol was not without its inventors; Plate XXIV illustrates a machine for the quantitative estimation of the knee-jerk devised by a house surgeon at the Children's Hospital.

Two events stand out. The first was the national meeting of the British Medical Association in Bristol in 1894 when Edward Long Fox was President. This is fully reported in the Journal.

The second event *almost* made medical history. On 8th November, 1895 (in the afternoon), Röntgen discovered X-rays. His findings were published with astonishing rapidity before the end of the year and were mentioned by the *Daily Chronicle* and the *Lancet* in January 1896. The first account in this country of the practical use of X-rays in surgery appeared in the *Lancet* of 22nd February, 1896. In the Bristol Medico-Chirurgical Journal of March 1896, the X-ray photograph shown in Plate XXV appeared together with the following report, in 137 words, of what but for a few weeks, would have been the first record in this country of the surgical application of Röntgen's discovery.

**Plate XXV—From Vol. XIV (1896). One of the first skiagrams to be published in this country.**

### The New 'Photography'

On February 10th Mrs. L. consulted Mr. T. F. Edgeworth on account of pain in the front of the right wrist, due, she thought, to a needle which had accidentally penetrated the skin. There was some tenderness on pressure, but nothing further could be made out. Before any exploratory incision was made, Professor Wertheimer, the Principal of the Bristol Merchant Venturers' College, very kindly made a radiogram by Röntgen's rays. It showed the needle quite distinctly. A few days subsequently, after an injection of cocaine, the needle was easily removed. The radiogram was of great assistance in showing that the needle was there, and also its exact position, so that an incision could be made just over the upper end of it.
This is the first case in Bristol in which surgical use has been made of the discovery.

A second X-ray photograph was published in the Journal six months later. By 1899 X-rays were in common use and William Cotton was describing a 'Simple form of Influence Machine for X-ray Work'—(Plate XXVI)—a sort of 'Do-it-yourself' kit needing only an intelligent small boy to turn the handle; the exposure time needed to demonstrate a healing fracture was however ten minutes. Judging from a number of similar articles which he wrote about this time, Cotton must have been an interesting character but I have been able

**Plate XXVI—'Simple form of Influence Machine for X-ray Work'. Vol. XVII (1899)**

to find out little about him. He qualified in 1883 and died about 1929 and was at one time medical officer to Bristol Prison. I imagine him as one of the original 'boffins' applying new ideas from science and technology to medicine—but I might be quite wrong.

In 1896 local correspondents for the Journal were appointed in Bath, Cardiff, Exeter, Gloucester, Newport, Plymouth, Swansea and Torquay. Then came the turn of the Century. The Journal appeared with a black border to mark the death of Queen Victoria. Photography was improving and photomicrographs of 'morbid growths' began to appear. A new cover design was introduced—and in 1902 Dr. Edward Long Fox died.

### Edward Long Fox

Edward Long Fox (Plate XXVII) was born in 1832, the son of Francis Ker Fox and grandson of the other Edward Long Fox who had served the Bristol Infirmary as physician for thirty years from 1786. After a short time at Edinburgh Medical School he became a student at St. George's Hospital, London, where he was at one time Clinical Clerk to Bence Jones. He graduated M.B. in 1857 and became M.D. in 1861. He was elected F.R.C.P. in 1870. When, like his grandfather and uncle before him, he was appointed physician to the Royal Infirmary in 1857, he displayed his characteristic tenderness and humility by saying to the students on his first visit—

> 'I wish to say that as I have only just passed out of the student stage myself, I shall feel greatly pleased should any of you notice anything overlooked in my walk and practice here that might be of importance in the treatment of cases if you would kindly remind me of the fact for by such means we shall be serving the patients as well as helping one another.'

**Plate XXVII—Edward Long Fox**

He was a loyal and true churchman and President of the National Temperance League. A good friend to students, he was also physician to Clifton College for twenty years from its foundation in 1862. His many honours and achievements have been recorded in countless Long Fox Lectures but the character that comes through it all is that of a truly good physician, well-loved throughout the city. In the early 1860s during a typhus epidemic in a poor part of Bristol he established a temporary hospital there but many of the sufferers would not leave their homes until Edward Long Fox went personally to persuade them.

### The Long Fox Lecture

When Edward Long Fox died at the age of 70 his many friends subscribed to a fund which was to be used for three purposes, (1) to erect a memorial brass in Bristol Cathedral, (2) to endow a fund to maintain a medical missionary student in Bristol University College and (3) to provide for a public Memorial Lecture to be given annually on a medical or allied scientific subject by a selected doctor who was to be from Bristol or its neighbourhood or a former student or member of the teaching staff of Bristol University College. The first such Lecture was given in 1904 by John Beddoe, formerly a physician to the Royal Infirmary.

The Bristol Medico-Chirurgical Journal has a special interest in the Lecture since it is a rule that if the Lecture is to be published it must be offered first to the Journal. In fact over the years the Journal has published most of the Lectures.

### 1900 - 1912

We must pass over this period quickly although it is not without interest. In 1905 there was John Dacre's presidential address on 'The Family Doctor'. In 1906 Walker Hall wrote on 'New Methods of Clinical Pathology'. In 1907 Carey Coombs (who was assistant editor for a time) contributed his first of many articles, this being on 'Rheumatic Carditis in Childhood'. In 1908 Munro Smith recorded a successful operation on the middle meningeal artery and in 1909 there was a report on an epidemic of smallpox in Bristol. 1910 brought comments on the new State Medical Insurance scheme and in 1911 Newman Neild wrote on 'Tea Poisoning'. By 1912 some of the articles begin to have a rather modern flavour—Walter Swayne on 'The Problem of Medical Education' and Odery Symes on 'The Heroin Habit'. The steady recording of cases and experiences continued as did 'Scraps'—

'Doctor: Does your little boy always stutter?
Mother: Oh, no Sir—only when he attempts to talk.'

New features included Editorial Notes, news, comments, examination results and reports of medical society meetings and the progress of the library.

The advertisements from this period also have their fascination and still brought in the money. At the end of 1912 Shingleton Smith retired after twenty years as Editor and the event was marked by a dinner and smoking concert at Fortt's restaurant.

### 'The Stethoscope'

At this point I must digress a little, to say something about another medical journal which had meanwhile made its appearance in Bristol. This was the medical students' journal 'The Stethoscope' which began publication in 1898 and appeared about every two months until 1927. Its first editor felt that

> 'Our highly respected Medico-Chirurgical Journal was too advanced and clever for the man starting his medical career and that we needed a magazine essentially our own, instructive and interesting to the average student, with an intermingling of the lighter element to enliven our weary hours.'

It published fairly simple medical articles together with some diagnostic puzzles with the answers in the next number. For the rest it consisted largely of reports of debates, sporting events, club activities, marriages, nursing appointments and so forth. There were also some good caricatures and much good humour—

1901: The Verb 'To Clerk'

Present: I am clerking
Thou messest about
He is our physician
We are his mere clerks, etc.

Future: I shall do it tomorrow
Thou wilt be had on toast
He will want visual fields, charts, etc.
We shall have no excuse next time.
Ye will look wise and say nothing.
They will alter their histories considerably.

Perfect: I heard breath sounds normal
Thou didst not know any better
He heard rhonchi, rales, crepitations amongst other things
We did not feel at all surprised
Ye heard everything he heard
They wondered what the deuce it meant.

It seems to have been edited and written mostly anonymously—for obvious reasons—but was well produced and makes very good reading even today.

**Patrick Watson Williams** (Editor 1913 - 1926)

The next Editor, Patrick Watson Williams (Plate XXVIII) was a Bristolian, educated at Clifton College and Bristol Medical School. He became assistant physician to the Royal Infirmary in 1888 and Physician to the Nose and Throat Department in 1905. A book which he published in 1892 gave him an international reputation and he became one of the great pioneer ear, nose and throat surgeons, being given the title of Consulting Surgeon in 1921. He was a good artist and etched his own Christmas cards; he was also interested in fishing and billiards. He married Edward Long Fox's daughter, Margaret.

**Plate XXVIII—Patrick Watson Williams. Editor 1913-1926**

As Editor he kept the Journal alive during the 1914-18 War, making sure among other matters that there were adequate stocks of paper. During the War the volumes got slimmer and as would be expected many papers are connected with wartime activities.

The intriguing William Cotton crops up again, this time describing a Field Service X-ray outfit in France. There were problems at home too; an article on Institutional Dysentery at Stapleton Workhouse (now Manor Park Hospital) appeared in 1915 and in the following year an account of Plague in Bristol. That same year saw an Obituary Notice for Dr. W. G. Grace, the great Gloucestershire cricketer.

Even after the war ended there were difficulties and the Journal does not seem to have got back to normal until about 1923. Patrick Watson Williams relinquished the editorship in 1926 after 25 years on the editorial committee and 13 years as editor.

**J. A. Nixon** (Editor 1927 - 1947)

His place was taken by J. A. Nixon (Plate XXIX) who had qualified from St. Bartholomew's Hospital (where he edited the Hospital Journal) in 1900. He came to

**Plate XXIX—J. A. Nixon. Editor 1927-1947**

the Royal Infirmary in 1902 and became full Physician there in 1909. He was interested in industrial diseases, especially dermatitis and was also one of the first physicians to use insulin in this country. He was an extraordinarily conscientious and precise teacher. Although then retired he would still come into the wards when I myself was a house physician and would question us young doctors closely on clinical details which sometimes seemed exasperatingly more important to him than they did, at the time, to us. This precision and attention to detail however was no fault in an editor and stood him in good stead when he found himself destined to keep the Journal going through a second war.

In 1933 a special number was published commemorating both the Centenary of the Bristol Medical School and the fiftieth Anniversary of the Journal.

The advent of chemotherapy is recorded in 1937 with articles on sulphanilamide and Prontosil.

Only two numbers were produced in each year 1941 and 1942 and only one number in each of the three following years. There were articles on air raid casualties and other wartime topics.

The Journal remained slim for several years after the war and in 1947 there was much discussion as to whether the Journal was still of value to the Society. It was decided that it was of value and would carry on. It would also try to extend its readership more widely in the West of England.

**'The Black Bag'**

During Nixon's editorship another contemporary journal appeared. This was the 'Black Bag' introduced as a medical students' journal in 1937, 'The Stethoscope' having ceased publication ten years previously. In many ways it has carried on the tradition established by 'The Stethoscope' but has had a rather more chequered career. The pre-war numbers were extremely well produced and followed the same pattern of contents—some medical articles by senior doctors together with student articles on travel, history and so forth. There were also humour, social news and book reviews. Of recent years it would seem that the magazine keeps changing its size and shape, is rather irregular in its publication and frequently seems to be making new starts. All this is probably an inevitable result of constant changes of youthful editors.

**Eric Watson Williams** (Editor 1948 - 1951)

Eric Watson Williams (Plate XXX), son of Patrick Watson Williams, was born in 1890. After graduating from Cambridge he almost immediately joined the R.A.M.C. and was awarded the M.C. after the Battle of Lys in 1918. He became a registrar in his father's department and became a full surgeon to the Ear, Nose and Throat Department of the Royal Infirmary in 1926. He was associated with the Journal for 30 years. He was well versed in classical studies and an antiquarian making a number of contributions to the literature. He was precise in his habits and a surgeon of great skill. He remained editor for only a short time, resigning in 1951. He was forced to make many

**Plate XXX—Eric Watson Williams. Editor 1948-1951**

economies in the Journal, dropping obituary notices, book reviews and library lists. He was very proud of his association with the Royal Infirmary and with the Journal and made great efforts to make it a Journal for the West of England.

**The Journal since 1951**

Since 1951 I shall have little to say. There will be no more thumbnail sketches of the editors; they are still alive and I will not embarrass them. The Editors since 1951 have been Mr. Keith Lucas, Dr. J. E. Cates, Dr. Alan Raper and myself.

In 1953 the name of the Journal was changed to 'The Medical Journal of the South-West'. Those who decided this had genuine motives and hoped that it would help to weld the new South-West Region of the National Health Service together. They were not to know that it would not work out that way and that loyalties are perhaps more local. Personally I always thought the new title was a mistake. It was so clumsy. If it had to be changed, 'The West of England Medical Journal' would have been much better. Many people were, I think, relieved when in 1963 the Journal resumed its old name. A new cover design then appeared. In recent years there has been a constant struggle to get either contributions or money—or both. Lack of copy distresses the Editor but tends to please the Treasurer, lack of money distresses both. In 1968-69 there was much questioning as to whether publication should continue. The Society decided it must go on and the Editorial Committee was strengthened. There was a boost in 1969 when the Symposium number commemorating the retirement of Professor C. Bruce Perry was published. In 1970 the Journal assumed a new shape and size and for the first time was printed in columns with illustrations in the text. This seems to have had some effect and authors are now queueing up to write in it. But money is running out and the price must go up. How we wish we could raise money by advertising as in the 1890s when it made so much money that the Society had to be restrained from giving it all to charity!

**The future**

It seems likely that that time has gone for ever and the Society will have to subsidise the Journal. So the whole question is raised again. Why keep it going? Is there still a place for a local medical journal? My answer to that is a definite 'yes' for the following reasons:—

1. It is a record of thought and practice in medicine in and around Bristol and is therefore a document of some historical value.
2. It should provide a good training ground for young writers who can be taught and encouraged by an Editor known to them who will not just reject their contributions out of hand as some more distant Editor might do.
3. It is listed in the Index Medicus and so all contributions become part of the 'world literature'.
4. It is sent all over the world in exchange for other journals which enrich our medical library. In 1884 there were 39 such exchanges, now there are no less than 120 including such publications as the British Medical Journal and The Practitioner.
5. It may be just because no-one can bear to see it die, and this is not just mere sentiment.

Perhaps Greig Smith's words in his circular sent out to all the practitioners in Bristol and the surrounding area still apply,

> 'Such a publication issued in our midst and started by ourselves would not only rescue from oblivion much that might otherwise be lost, but would also stimulate us more keenly in our efforts to advance medical science.'

I am sure that 80 years ago a speaker schooled in the classics, which I am not, would have ended this lecture with a parody in Latin of that quotation from Lucilius which the Journal used to bear on its cover. Perhaps I might attempt a parody in English—

*Even our little local knowledge is ignorance unless that knowledge is made known to others.*

**REFERENCES**

SKERRITT, E. M. (1892): The teachings of failure. This Journal **10**, 229-249.

SMITH, P. SHINGLETON (1900): Quoted in report on Presentation to Mr. L. M. Griffiths. This Journal **18**, 98.

## Figures and Tables

**Plate XIII f1:**
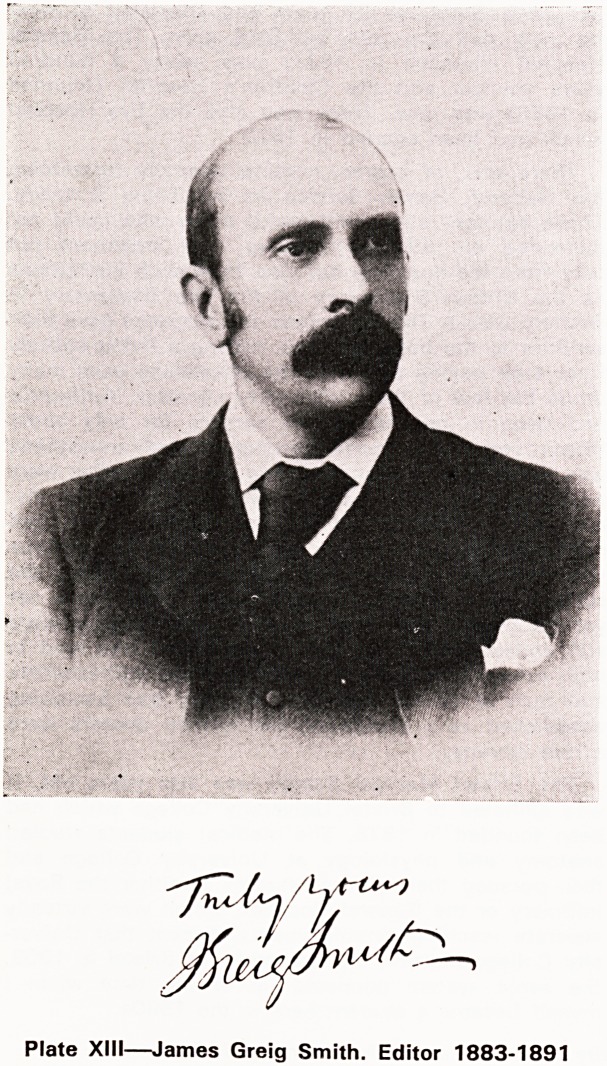


**Plate XIV f2:**
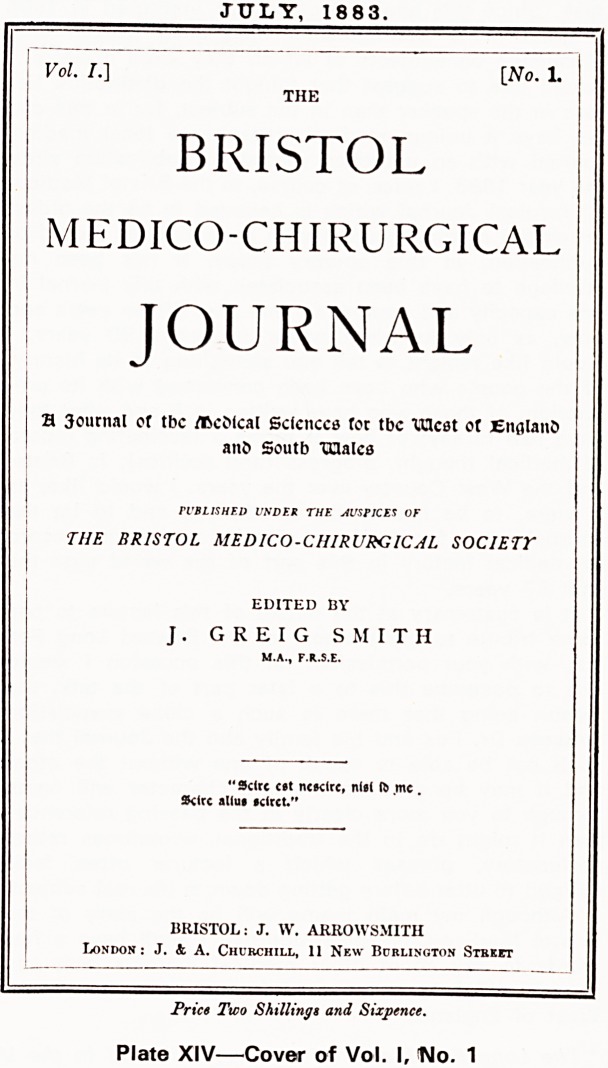


**Plate XV f3:**
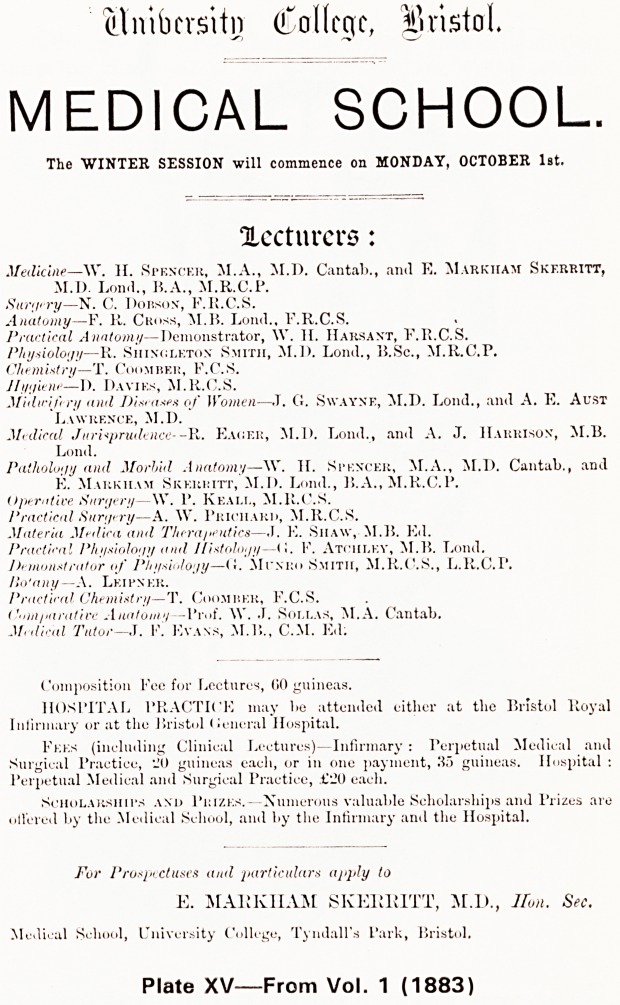


**Plate XVI f4:**
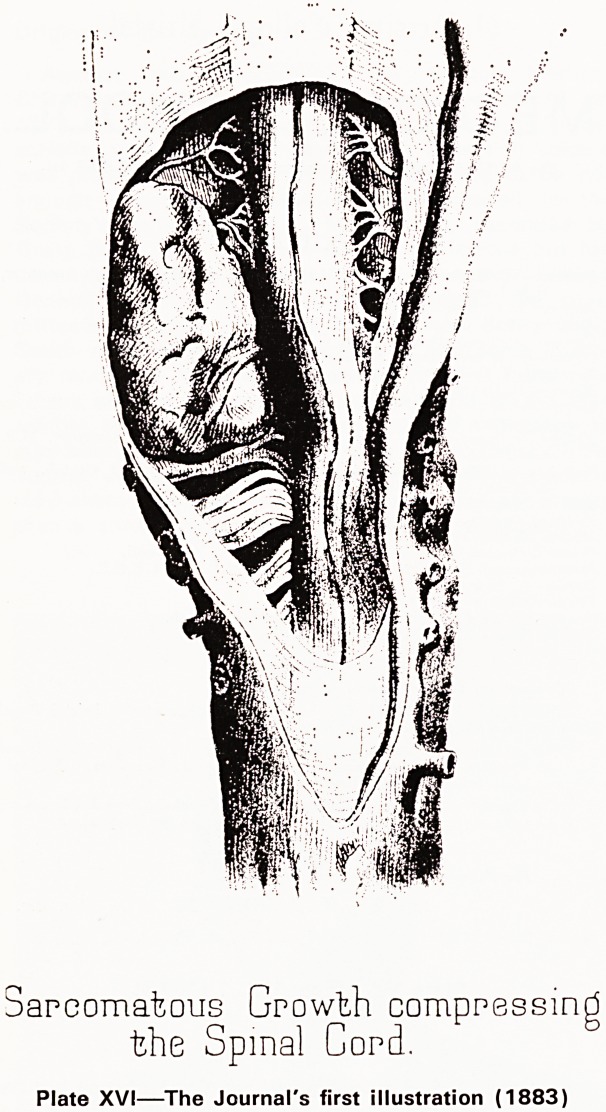


**Plate XVII f5:**
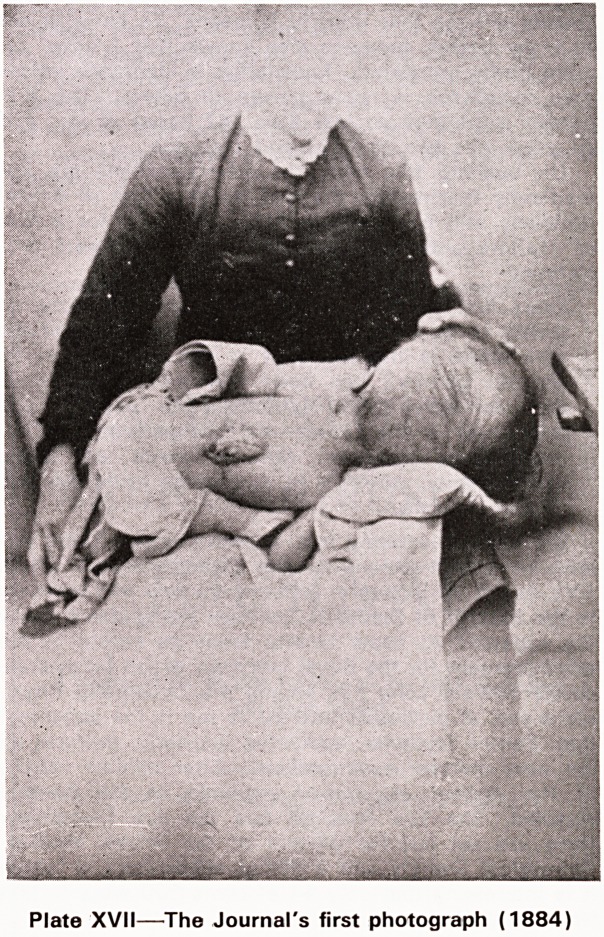


**Plate XVIII f6:**
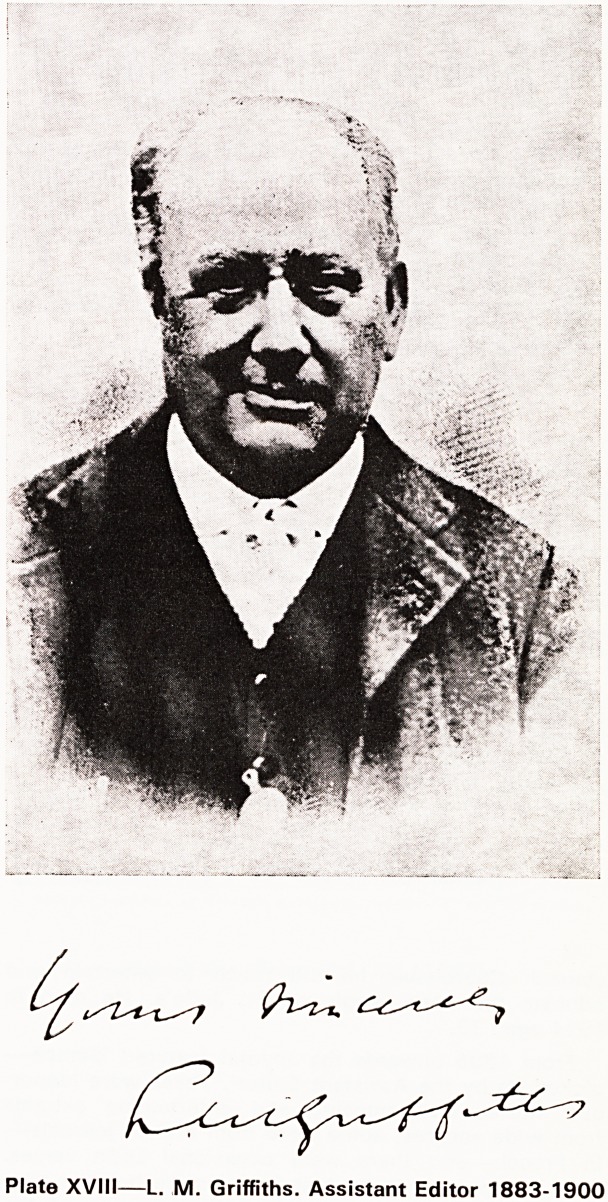


**Plate XIX f7:**
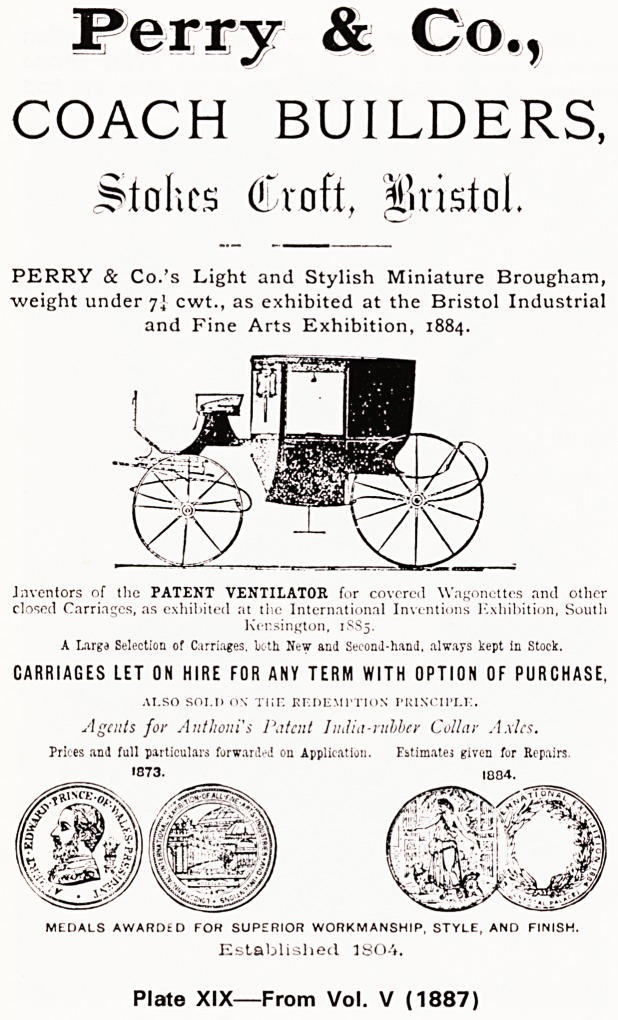


**Plate XX f8:**
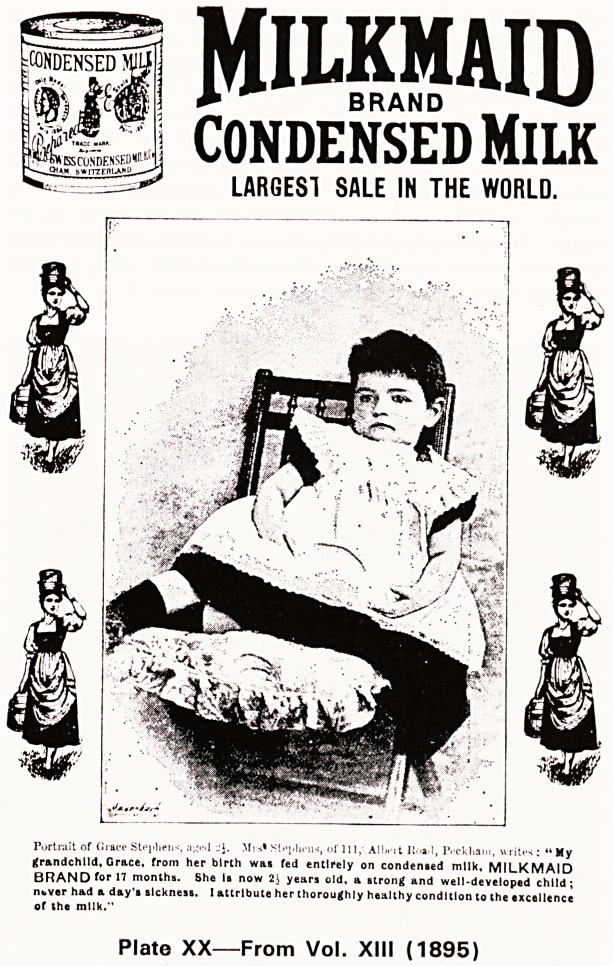


**Plate XXI f9:**
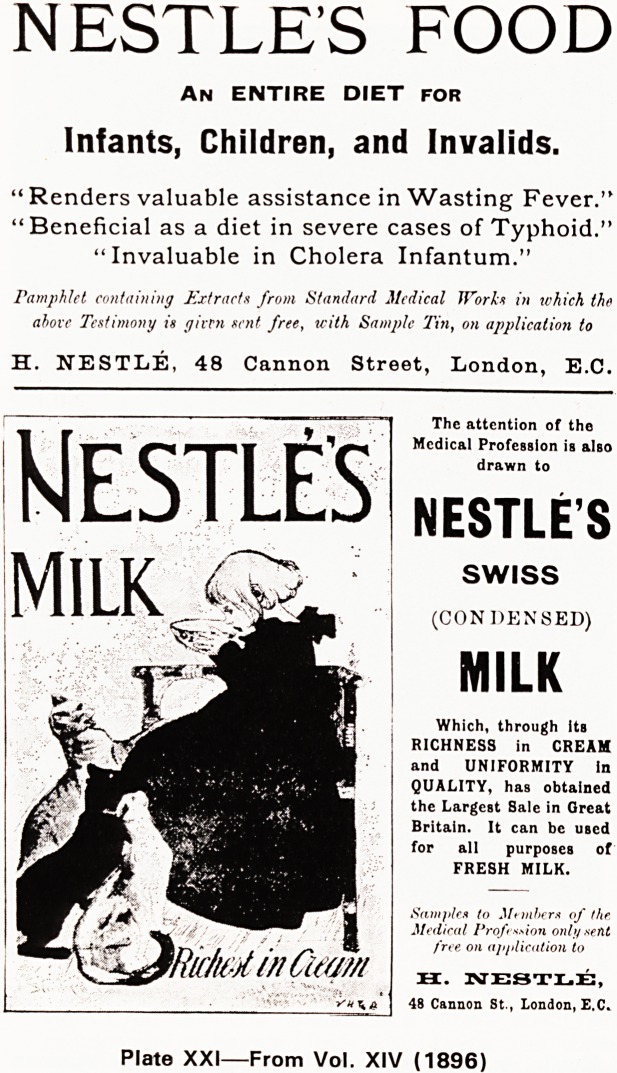


**Plate XXII f10:**
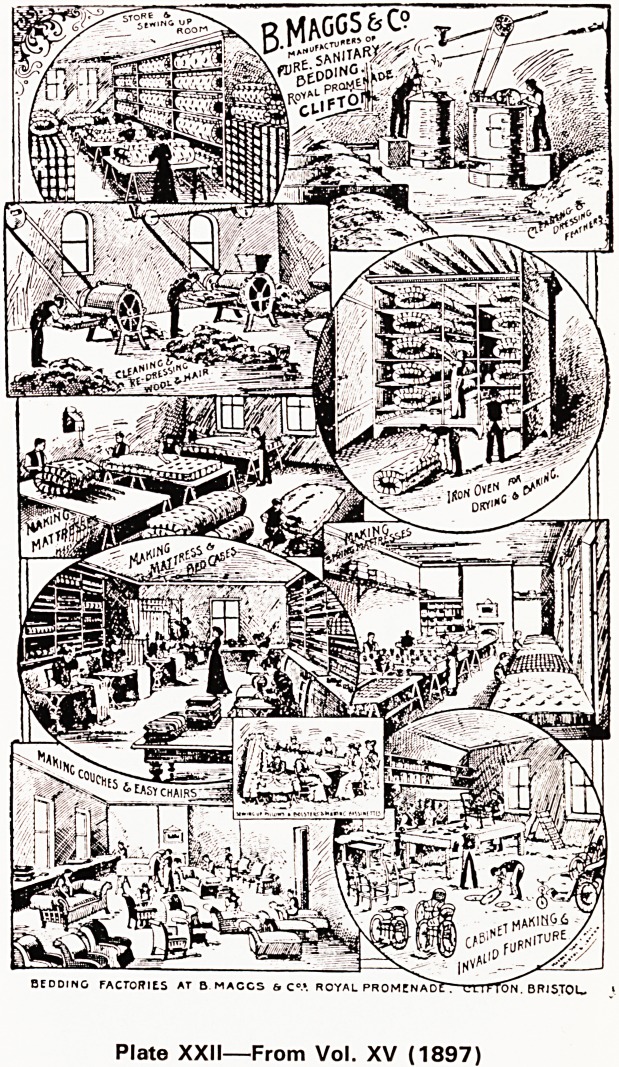


**Plate XXIII f11:**
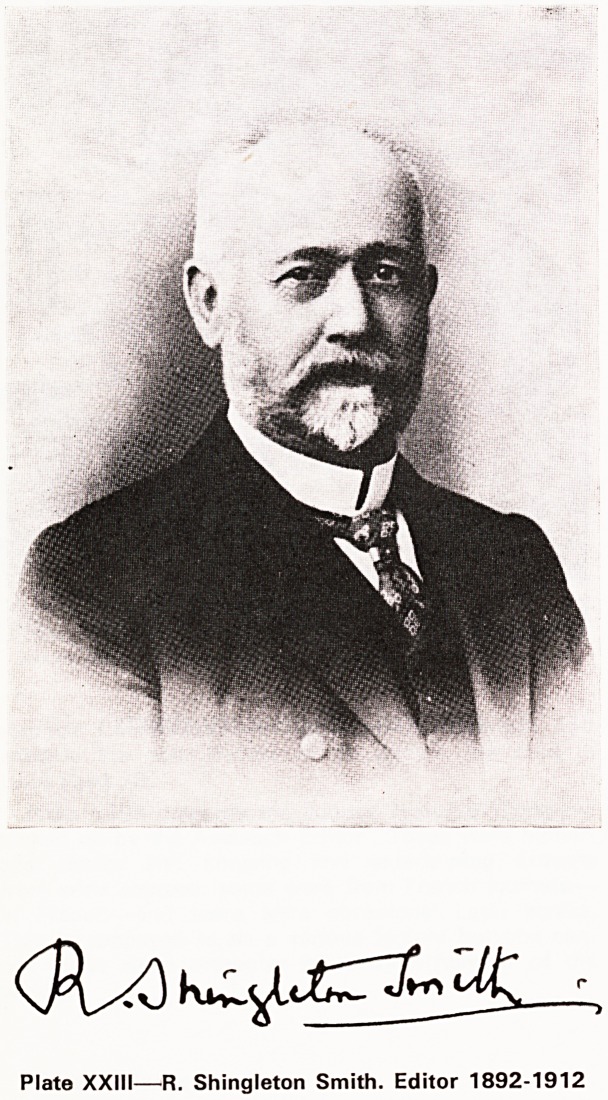


**Plate XXIV f12:**
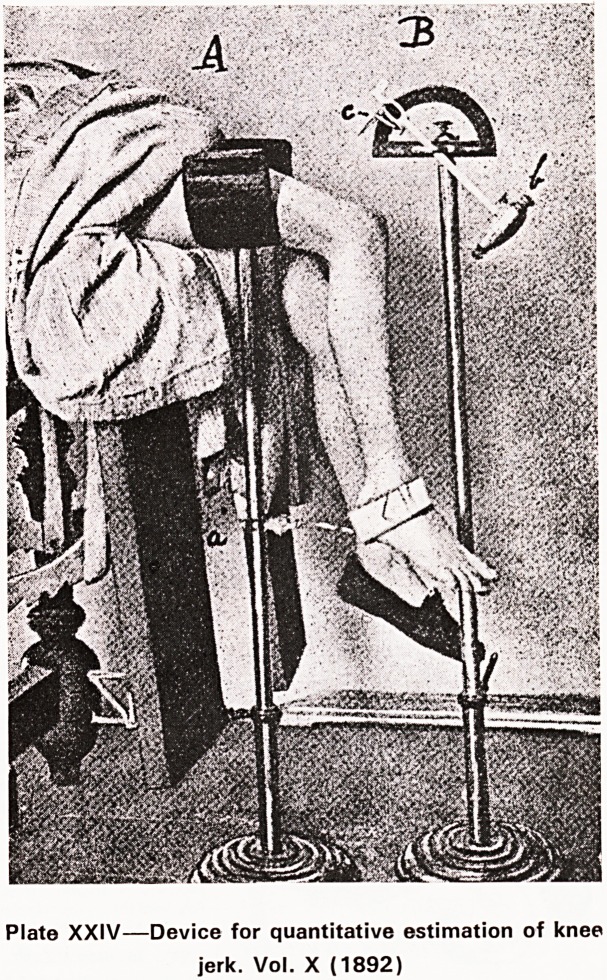


**Plate XXV f13:**
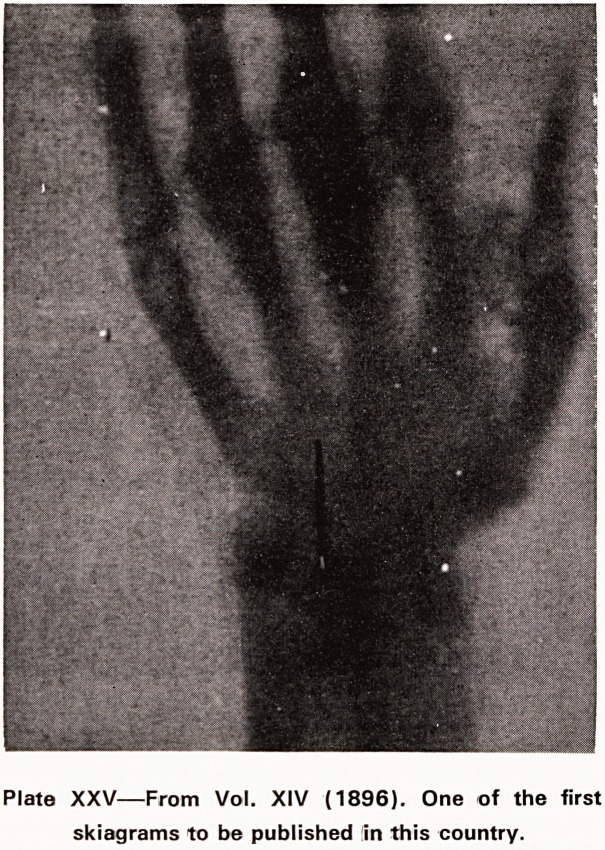


**Plate XXVI f14:**
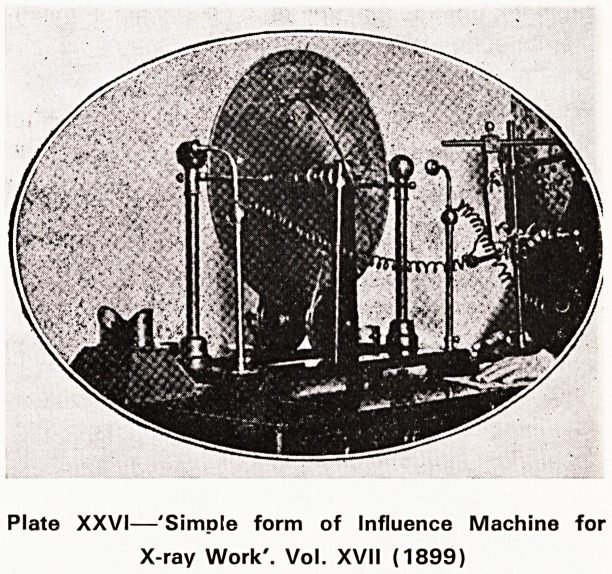


**Plate XXVII f15:**
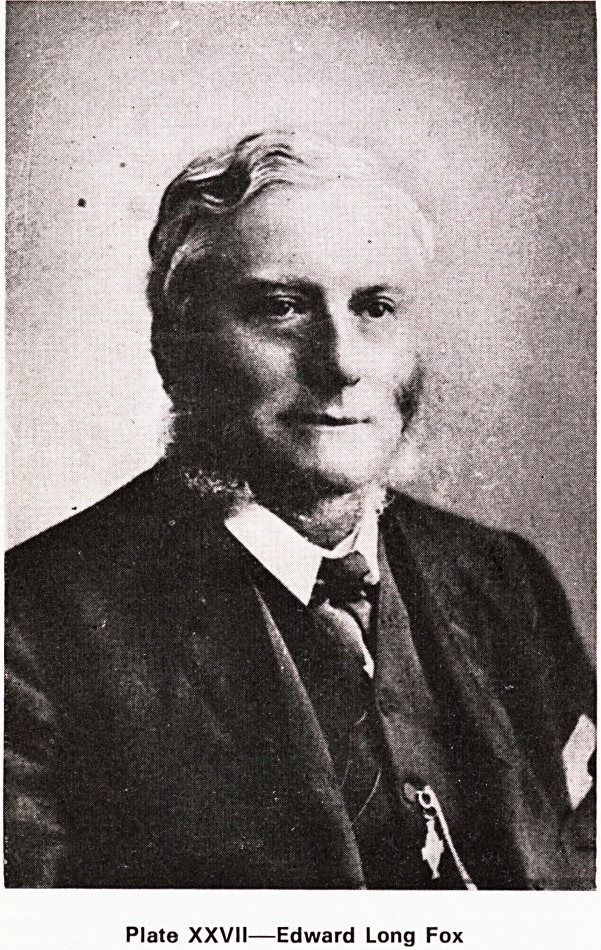


**Plate XXVIII f16:**
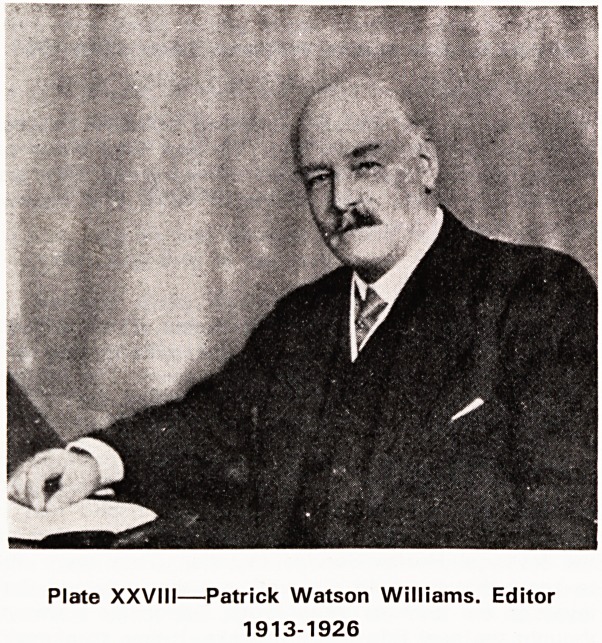


**Plate XXIX f17:**
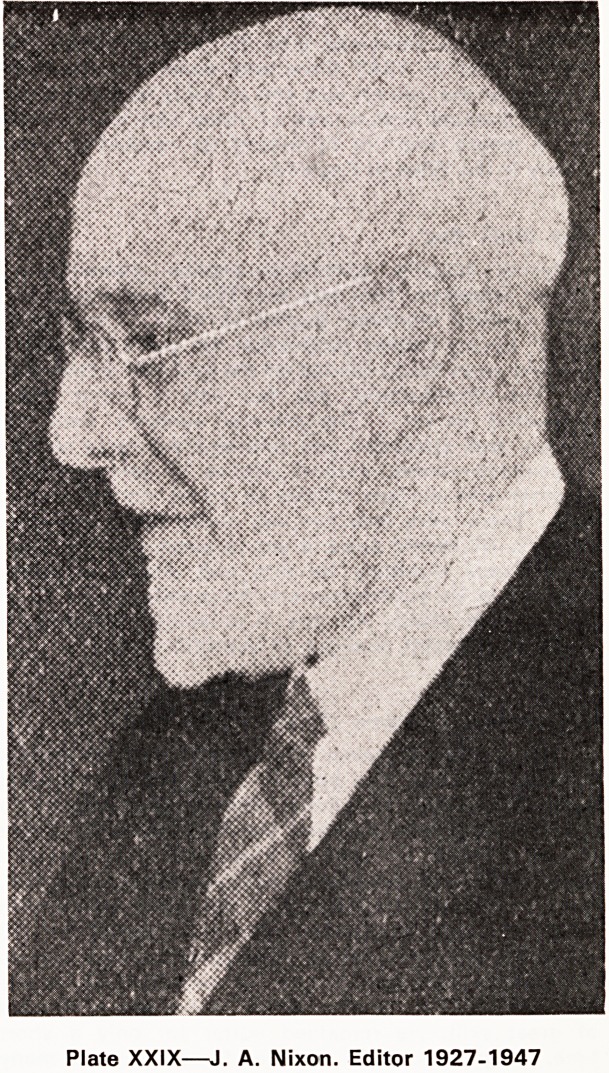


**Plate XXX f18:**